# Kansas’ Mammoth Cave

**Published:** 1881-09

**Authors:** 


### KANSAS' MAMMOTH CAVE.

### DISCOVERY OF A CAVE CONTAINING MANY ABORIGINAL REMAINS.

The discovery of the cave was made about a month since. Thomas county being only sparsely settled, the discovery was accidental and made while following a wounded wolf, who took refuge there, and finding a human skull and other bones, from which the flesh had long since been removed at its entrance. The discoverer, supposing it to be a wolf's den, obtained the assistance of Mr. Hamilton, who happened to be in the vicinity, and the owner of the land, and the three, well armed and carrying a lantern, proceeded to the spot at the base of a high bluff, and pushing aside the underbrush found an opening in the ground, irregular in shape and about three feet in diameter. Into this opening the party cautiously proceeded on their hands and knees a distance of three or four feet, when the passage way, enlarging in every direction, permitted them to assume an erect position, and they found themselves in an irregular-shaped room, its ceiling sloping upward and out of sight. The place was intensely dark, only a few rays of sunlight penetrating through the entrance, and the light of the lantern seemed but to make the darkness more perceptible. Passing over the wolf's body they found that the floor of the room was covered with human skeletons and bones entirely denuded of flesh, placed in every conceivable position ; some stood upright against the walls, others were in a sitting posture, but the greater part lay scattered on the floor in confused masses. The room was somewhat triangular in shape, its longest side being upward of forty feet and the others about twenty each. Hung on its walls or resting against them, and lying on the floor among the skeletons, were numerous shields and spears and other implements of warfare of a savage race.

On the next morning, having procured two additional lanterns and improvised a torch, the explorers re-entered the cave, and, clambering over the skeletons to the aperture in the wall noticed the day previous, they entered it and found themselves in a passage-way about four feet high and ten feet wide, and arched overhead; the walls were of solid white rock and covered with moisture. The floor of the hall-way sloped downward. The hall-way was about twenty feet long, and appeared to have been cut in the solid rock by skilled workmen. At the further extremity it opened into another chamber, on the threshold of which the party was halted by noises resembling the movements of animals within. Peering into the Egyptian darkness and discerning nothing and thinking that they had found the wolf's abode, one of them fired his pistol, and their ears were astounded with a succession of reports as from a dozen pistols, repeated from all parts of the room. An involuntary exclamation of surprise escaped from one of the party, and his words, "Great God!" were in a like manner distinctly echoed and re-echoed as by a dozen voices in as many tones, finally dying out, apparently, in the far distance.

After their astonishment had subsided they were convinced that they had discovered a veritable echo gallery. Leaving a light at the entrance to mark its locality, they proceeded to explore it, and found it to be nearly circular in form and nearly one hundred feet in diameter. The walls were perpendicular and rising to a great height and having numerous niches, some apparently being openings into other chambers ; the lights were not strong enough to reveal the ceiling. The floor was solid rock and quite level and smooth and very damp. On one side of the room was found a platform of solid rock about twenty feet square, rising abruptly from the floor to a height of about four feet, otherwise the room was quite empty. Mr. Hamilton here said emphatically : " Nature might have furnished the outlines of the cave, but nature never uses a square in its work and never makes right angles, and never chisseled that platform nor the hallway through which we entered the room—it was the work of flesh and blood."

On the other side of the platform was found two openings in the walls, one of which resembled the hallway between the two chambers, but with its floor obstructed with masses of rock. The other was an arched way about twenty feet high, the arch extending to the floor. Passing through the arch a short distance, they came to a stream of water about a foot in depth, passing over a bed of white sand. The water was very clear and cold, and, though evidently flowing, had but little velocity. The width of the stream could not be determined, but as far as the lights could penetrate it, was arched over by solid walls of rock, approaching very near its surface.—*Gaylord* (Kan.) *Herald.*

